# Tissue gene expression analysis approach in a context of high technical and biological heterogeneity

**DOI:** 10.21203/rs.3.rs-6116991/v1

**Published:** 2025-04-07

**Authors:** Evan Bagley, Souvik Seal, Lauren R. Fanning, Jean-Sebastian Anoma, Tami Crawford, Benjamin G. Vincent, Elizabeth L. Barry, Martha J. Shrubsole, Erin Kirk, John A. Baron, Dale C. Snover, David N. Lewin, Todd A. Mackenzie, Xiaohua Gao, Melissa A. Troester, Alexander V. Alekseyenko, Kristin Wallace

**Affiliations:** Medical University of South Carolina; Medical University of South Carolina; Medical University of South Carolina; Medical University of South Carolina; Medical University of South Carolina; University of North Carolina at Chapel Hill School of Medicine; Dartmouth Geisel School of Medicine; Vanderbilt-Ingram Cancer Center; University of North Carolina at Chapel Hill; University of North Carolina at Chapel Hill School of Medicine; Fairview Southdale Hospital; Medical University of South Carolina; Dartmouth Geisel School of Medicine; University of North Carolina at Chapel Hill School of Medicine; University of North Carolina at Chapel Hill School of Medicine; Medical University of South Carolina; Medical University of South Carolina

**Keywords:** immune expression, NanoString, sessile serrated lesion, RUV

## Abstract

**Background:**

Immune expression profiling in colorectal lesions may provide insights into the origins of antitumor immunity and senescence. Optimal approaches for analyzing samples with lower quality RNA from molecularly diverse lesions are lacking. Therefore, we developed a NanoString nCounter-based approach for quality control (QC), normalization, and differential expression (DE) analysis, optimized for FFPE samples in contexts of high biologic heterogeneity.

**Methods:**

The approach incorporates a colon specific positive control gene set (11 genes) to minimize sample exclusions. We evaluated three normalization methods Removal of Unwanted Variation (RUVg), NanoStringDiff (NSDiff), and nSolver using a 277 gene immune panel to compare 100 samples, including sessile serrated lesions (SSLs) (n = 25), tubulovillous and villous adenomas (TVs) (n = 27), and tubular adenomas (TAs) (n = 48) We assessed Type I error rates, computational efficiency, and gene significance via FDR-corrected q-values.

**Results:**

Incorporating the colon-specific QC set reduced sample exclusions by 63% compared to standard methods (13 vs 35 sample exclusions). All three normalization approaches identified DE genes between SSLs and TAs (e.g., TFF1, MUC5AC, MUC6). For TVs vs. TAs, only RUVg and NSDiff detected significant DE genes, revealing wide-spread under-expression of innate and adaptive genes. While NSDiff labeled twice as many significant genes as RUVg, suggesting greater sensitivity, it also exhibited higher Type I error rates and increased computational demand.

**Conclusions:**

RUVg achieved a balance between computational efficiency and low Type I error, while NSDiff was more sensitive but computationally demanding and exhibited higher Type I error. Our approach provides a robust framework for profiling immune genes in heterogeneous lesions.

## Introduction

A robust immune response within the tumor microenvironment (TME) leads to a significantly reduced recurrence and mortality in colorectal cancer (CRC),^1–3^ but much less is known about the immune reaction in early carcinogenesis.^4^ Preinvasive lesion types exhibit molecular and pathologic heterogeneity,^5,6^ and emerging data suggests variable immune responses across lesion types as well. For example, sessile serrated lesions (SSLs) seem to display robust cytotoxic immune responses,^4,7^ whereas conventional villous-containing adenomas (TVs) appear immunosuppressive^8^ – resembling immune profiles of their invasive counterparts, microsatellite instability high CRCs and microsatellite stable (MSS) CRCs, respectively.^9,10^ Although gene expression profiling in preinvasive lesions may provide insights into the origins of anti-tumor immunity, or immune senescence, poor quality RNA coupled with biologic heterogeneity^11–13^ can pose challenges for differential expression (DE) analyses.^13^

Over the past decade, the NanoString nCounter platform has become a preferred technology for large-scale studies to assess immune gene expression in cancers because of its high precision and sensitivity, particularly for formalin fixed, paraffin-embedded (FFPE) tissues.^14,15^ Standard quality control (QC) procedures adapted for this NanoString platform^16,17^ help remove unwanted technical variation (RUV) by excluding lower quality samples before normalization and DE analyses. Typical QC steps^16^ include assessing experimental errors (e.g., binding density, imaging quality, level of detection), identifying outliers, and assessing gene missingness usually defined as > 30% of endogenous and ≥ 20% of housekeeping genes within a codeset (set of genes assessed in an nCounter study) falling below the background threshold. However, in datasets where missingness may reflect biologic phenomenon such as widespread immune suppression or low cellular heterogeneity, discarding samples with high missingness has the potential to introduce bias. Alternative QC approaches^17^ use biologically relevant positive control genes (e.g., cancer-specific markers) to exclude only samples with high missingness in the positive control genes.

Despite advances in QC and normalization methods for nCounter, there is limited guidance for expression analyses in the context of a wider range of sample quality common in FFPE tissues (e.g., low RNA yields, high fragmentation, especially associated with older specimens and high biological heterogeneity common in studies comparing molecular phenotypes or histologic types. To address this gap, we present a robust approach for gene expression analysis using a custom codeset of 277 immune-related genes, including 11 colon-specific positive control genes, and 15 housekeeping genes (Table 1).

## Methods

In the pre-normalization phase we apply principal components analysis (PCA) using the interquartile range (IQR) method to identify outliers in both Principal Component 1 and Principal Component 2 as previously described.^2^ We then assess missingness within our 11-gene colon-specific positive control gene set following an established protocol.^16^ We define missingness as >30% of the genes within our 11-gene set positive control set with counts below the background threshold (i.e., the average of the nCounter negative controls). After quality control we performed DE analysis across three heterogenous lesion types: sessile serrated lesions (SSLs), tubulovillous adenomas (TVs), and tubular adenomas (TAs). We compare three normalization methods (RUVg, NSDiff, nSolver) to evaluate the number of genes designated as differentially expressed at FDR-corrected q < 0.05 for SSLs. vs. TAs, and TVs vs. TAs. We then examine the correlation of log fold changes across the three methods, assess their proclivity for Type I errors via simulation, and report the computational time for the DE analyses.

This study is a cross-sectional analysis using a NanoString nCounter platform to assess differential gene expression in three types of preinvasive colorectal lesions (SSLs, TVs, TAs) using a custom immune gene panel of 277 genes. For this study, a random sample of 100 colorectal adenomas and serrated lesions was chosen from a larger multi-site retrospective study on immune contexture in colorectal adenomas and serrated lesions. The samples were from participants from three completed investigations: Vitamin D/Calcium Polyp Prevention Study (clinical trial, 2008–2015, ClinicalTrials.gov number, NCT00153816),^18^ Diet and Health Studies III-V (cross-sectional study with follow-up, 1998–2010),^19,20^ and Tennessee Colorectal Polyp Study (cross-sectional study with follow-up, 2003–2015)^21^. All subjects provided consent for participation in the original research studies. All research was performed in accordance with the Declaration of Helsinki. Data was deidentified prior to shipment to the data coordinating center at the Medical University of South Carolina. All research performed at MUSC was approved by the IRB-II -Medical University of South Carolina as exempt. All participants were diagnosed with one or more histologically confirmed colorectal adenomas or SSL at the start of the study by local GI study pathologist. Exclusion criteria included a history of colon resection, colorectal cancer, inherited colon cancer syndrome, or inflammatory bowel disease.

The NanoString nCounter platform analyzes up to 12 samples on a cartridge per run Prior to the start of the large study, we developed a stratified randomization scheme to minimize bias from potential batch effects. The randomization scheme assigned each sample to one of four strata: small (<10mm) TAs, large (10mm+) TAs, TVs, or SSLs. Each clinical site contributed samples to each stratum to fill 10 of the 12 positions within a cartridge. Given the higher prevalence of small TAs (~2:1) compared to large TAs, TVs, and SSLs, each cartridge typically contained four small TAs, two SSLs, two TVs, and two large TAs. Each cartridge also included two control samples: one replicate or pooled normal control (autopsy) and a Stratagene universal human reference (UHR), which is a pooled sample of 10 cancer cell lines (Agilent Technologies). RNA was isolated from two 10-micron thick FFPE samples using Qiagen ALLPrep Dual RNA-DNA isolation kit.^22^ Sample purity was evaluated using the 260/280 ratio and 260/230 ratio, and fragmentation was evaluated using the DV200 measurement on a subset of cases; however, these measurements were not used to exclude cases in order to minimize bias.

### Technical Quality Control Approach:

Prior to the start of the larger study, we conducted a small pilot (n=35) using traditional RUV-QC protocols.^16^ We found that 12 cases (35%) were flagged for exclusion due to high missingness in the endogenous and housekeeping probes. Given this high failure rate attributed to endogenous gene missingness, we adopted a modified QC approach for biologically heterogenous samples^17^ to be used in the present analysis. We selected 11 genes representing universal colon-specific expression (Table 1) to define our missingness flag. These genes were chosen by investigators with expertise in colorectal neoplasia across basic, clinical, and population sciences. The missingness was defined as 4 or more genes of the 11 genes in the panel which had expression levels below the average of a subject’s negative control probes. The negative control probes are technical probes included in every nCounter cartridge (NanoString, Gene Expression Data Analysis Guidelines (NanoString, MAN-C0011–04)). These control probes are designed against engineered RNA sequences which are not present in biological samples. Negative control probes are commonly used to set background thresholds.

The NanoTube^23^ *R Bioconductor* package was used throughout the workflow. First, we identified samples with extreme values (outliers) using a principal components analysis (PCA) and the IQR method in both PC1 and PC2.^16^ Extreme values were assessed visually on the PCA plot. Next, we excluded samples with missingness in the 11-gene colon-specific genes, as described previously.

### Choice of Normalization Method:

After technical QC, as described above, we consider three different normalization approaches: RUVg, NSDiff, and nSolver.^13,17,24^

#### RUV Approach:

RUVseq, an *R Bioconductor* package, offers several normalization techniques. For our study, we selected RUVg which uses housekeeping genes to estimate and subtract technical variation from the genes of interest, producing normalized expression data. Bhattacharya et al.^16^ adapted RUVseq for nCounter data by incorporating positive and negative controls into each NS cartridge. Our approach, as described previously^16^, is an adaptation of this protocol.

#### NanoStringDiff:

NSDiff, another *R Bioconductor* package, is tailored for differential expression (DE) analysis of nCounter data. The method jointly performs data normalization and DE analysis. Following the nCounter protocol, it normalizes the data using a positive size factor (positive control genes), background noise correction (negative control genes), and housekeeping size factor (housekeeping genes)^13^. In the DE analysis, it accounts for the over-dispersed nature of counts using a generalized linear model of the negative binomial family with empirical Bayes shrinkage.

#### nSolver:

Developed by NanoString Technologies, nSolver performs comprehensive gene expression analysis with normalization based on positive control genes and housekeeping genes, as per the Gene Expression Data Analysis Guidelines.^13^

### Differential Expression Analysis:

We present the results for RUVg, then compare these results with the other normalization approaches. All analyses focus on over- and under-expression of genes for SSLs vs. TAs (referent group) and TVs vs. TAs (referent group). For each method, we determined the estimated number of DEGs, correcting for multiple testing using Benjamini-Hochberg correction at q < 0.05.^23^ After normalization, we performed DE analysis across the three lesion types. RUVg normalized data can be fitted using a GLM or log-transformed and tested using a linear model and t-test.^17^ Instead of t-tests, following a newly developed *R Bioconductor* package NanoTube,^20^ we consider the log-transformed gene expression data from all the above methods and fit a multi-level linear model implemented in limma.^21^ We compared the DE analysis results from the three approaches: i) a multi-level linear model in limma on log-transformed RUVg-normalized data, ii) NanoStringDiff, and iii) multi-level linear model in limma on log-transformed nSolver-normalized data.

We then examined the correlation of log-fold changes among the methods using scatterplots To evaluate the Type I error for each normalization method, we conducted a simulation study to determine the empirical Type I error rate by randomly permuting the label for lesion type, normalizing the data and conducting DE analysis. The estimated Type I error rate was calculated as the mean of the number of significant DE analysis results (at alpha 0.05), which were represented as a binary variable (1=yes, 0=no). We replicated this 1000 times each for RUVg and nSolver normalization and 100 times for NSDiff normalization, due to the exceedingly slow computation of the NSDiff method. Finally, we report the computational time of each procedure.

## Results

**Figure 1:**
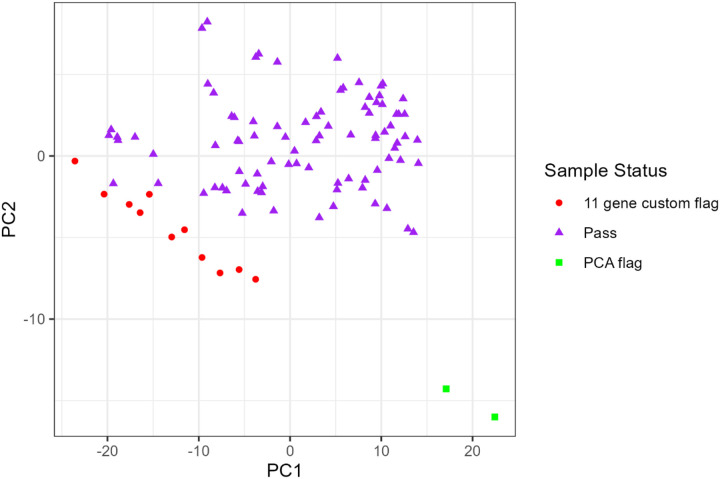
Principal component analysis (PCA) plot of 100 initial samples with corresponding pass or flag status.

We processed 12 cartridges with a total of 100 samples, 4 replicate samples, 6 normal tissues, and 10 UHRs. The dataset consisted of 25 SSLs, 27 TVs, and 48 TAs (Table 2). Analysis of the 4 technical replicates resulted in a correlation coefficient of approximately 0.99 across all endogenous probes. We excluded normal autopsy or UHR controls from this analysis. QQ analysis excluded 13 samples overall; PCA analysis identified 2 samples for removal due to extreme values and 11 samples were flagged for high missingness in the 11-gene colon-specific flag, leaving 87 samples for the DE analysis (Fig. 1).

**Figure 2:**
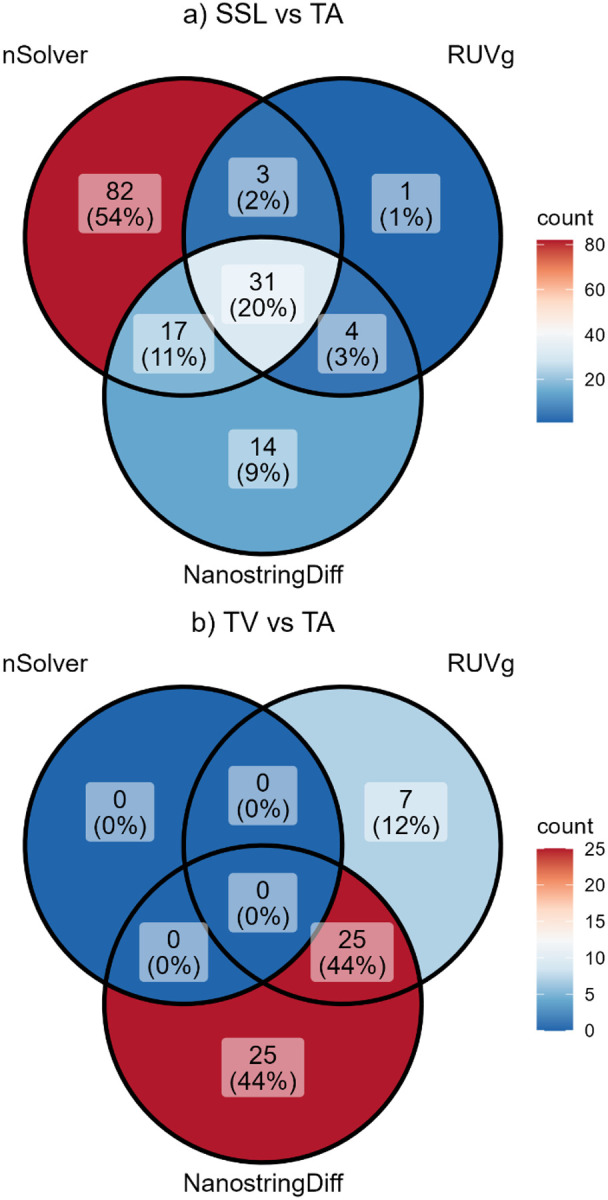
Venn diagrams of significant DEGs for nSolver, RUVg, and NSDiff normalization methods in **a)** SSLs vs. TAs, and **b)** TVs vs. TAs.

**Figure 3:**
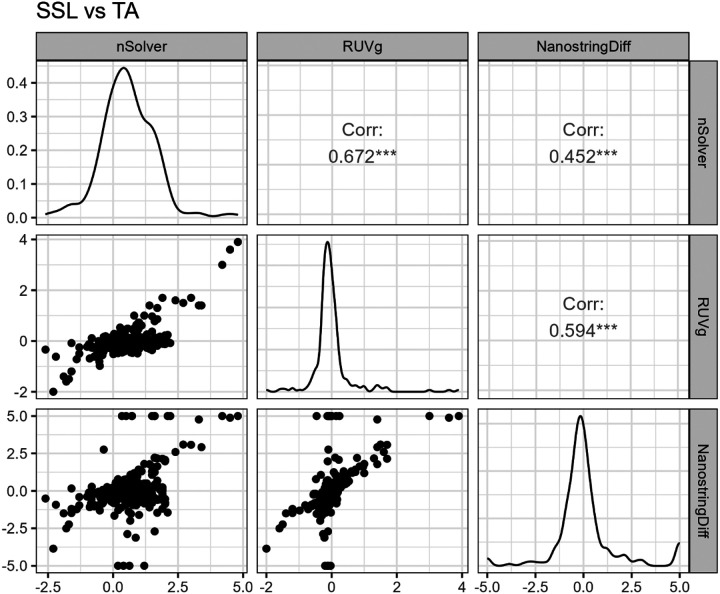
Correlation plot of log-fold changes (Log2FC) for SSLs vs. TAs using nSolver, RUVg, and NSDiff normalization methods.

**Figure 4:**
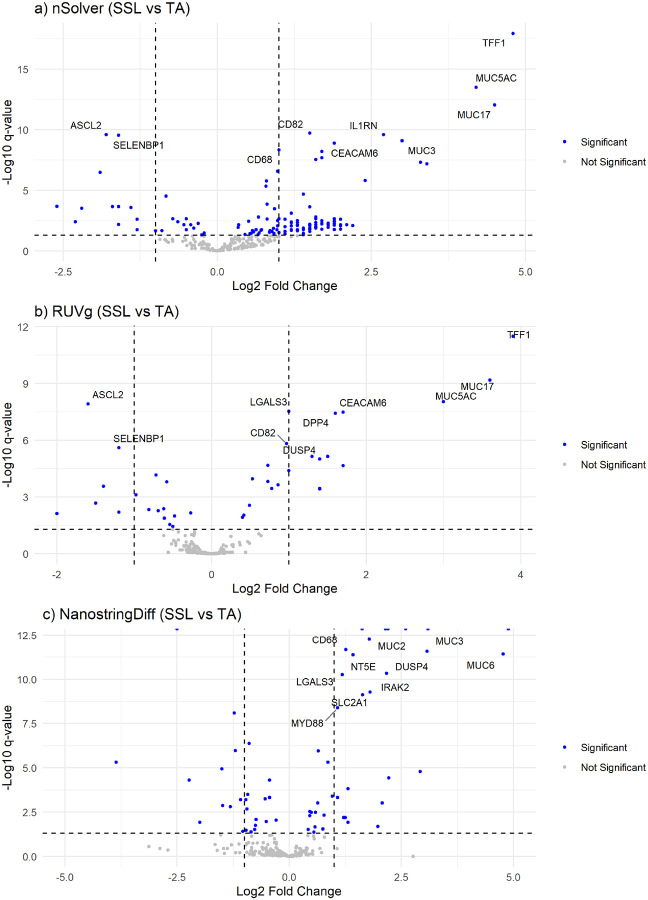
Volcano plots for SSLs vs. TAs showing Log2FC and −Log_10_(q-values) (significant DEGs shown in blue) for a) nSolver, b) RUVg, and c) NSDiff normalization methods.

SSLs vs. TAs: In the comparison of SSLs with TAs, all three normalization methods identified significant DEGs at a corrected q < 0.05. The RUVg labeled 39 genes as significant differentially expressed, NSDiff 66 genes, and nSolver 133 genes. (Fig. 4) NSDiff yielded several genes with extreme values, including 9 genes exceeding a p-value of 1.0 × 10^−16^ but small logFC values (Fig. 4). Thirty-one genes were common across all three methods (Fig. 2a). Correlation analysis of fold-changes indicated moderate correlation between RUVg and nSolver (r = 0.67) and between RUVg and NSDiff (r = 0.59). The correlation between nSolver and NSDiff was lower (r = 0.45) (Fig. 3

**Figure 5:**
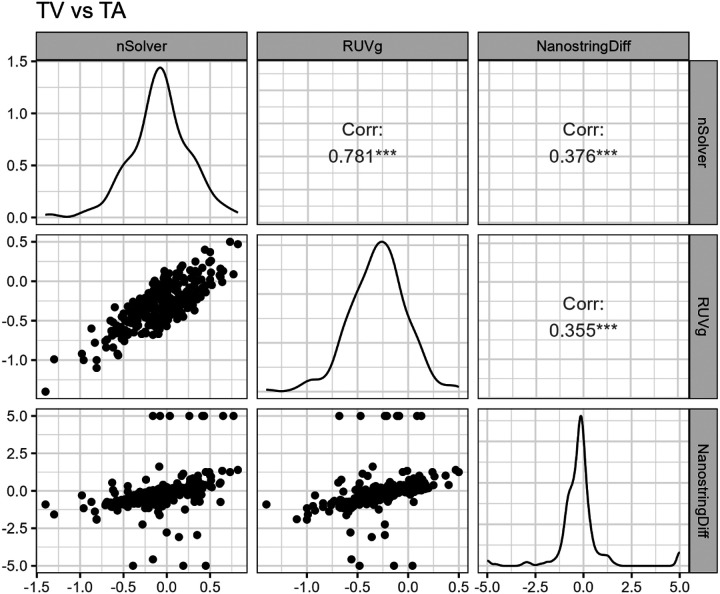
Correlation plot of log-fold changes (Log2FC) for TVs vs. TAs using nSolver, RUVg, and NSDiff normalization methods.

TVs vs. TAs: In the comparison of TVs and TAs, nSolver did not yield any significantly differentially expressed genes at q < 0.05. The RUVg method identified 32 DEGs and NSDiff identified 50 DEGs, with 25 in common (Fig. 2b). Correlation analysis among the fold changes indicated a poor correlation between NSDiff and RUVg (r = 0.37) and between NSDiff and nSolver (r = 0.35). A higher correlation was observed between nSolver and RUVg (r = 0.78) but also used limma methods post-normalization. Scatterplot of fold-change correlations for NSDiff and both other methods showed several aberrant points at the floor and ceiling, pointing to Type I error inflation or deflation (Fig. 5).

**Figure 6:**
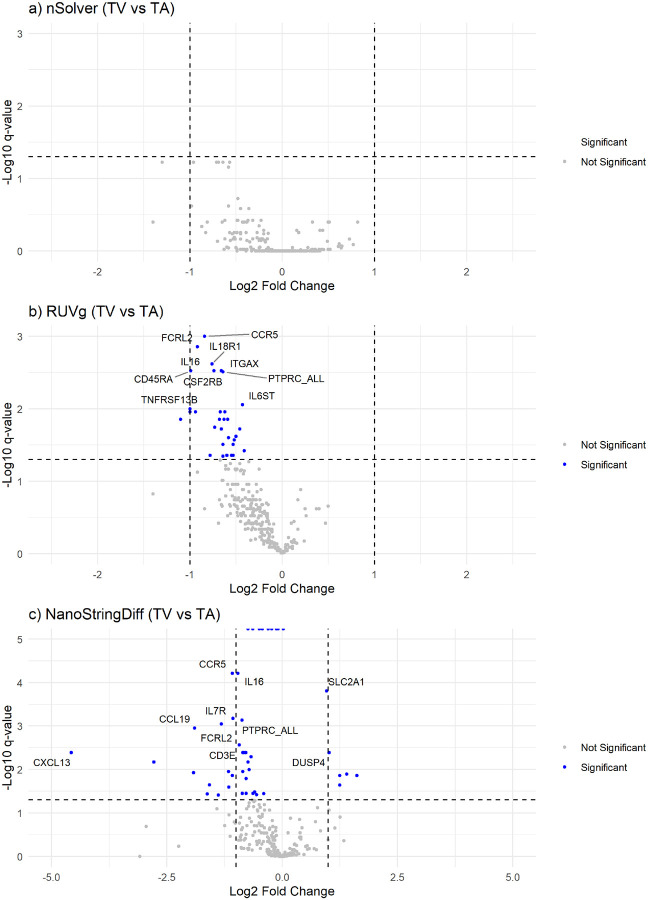
Volcano plots for TVs vs. TAs showing Log2FC and −Log_10_(q-values) (significant DEGs shown in blue) for a) nSolver, b) RUVg, and c) NSDiff normalization methods.

Volcano plots demonstrate widespread under-expression of genes in TVs compared to TAs for RUVg and NSDiff (Fig. 6). NSDiff labeled more genes as significantly under-expressed in TVs vs TAs than RUVg, however some of these genes had tiny log-fold differences (e.g., *BST1, STAT1, BRAF, STAT5A, RAF1*). Compared to RUVg, which had no over-expressed genes in TVs vs. TAs, NSDiff identified 9 genes which were over-expressed in TVs vs. TAs, including several mucin related genes (*TFF1, MUC5AC, DUSP4*) and genes related to altered glucose metabolism (*CCL4, SSP1, SLC2A1*).

### Shared DEGs

**Figure 7:**
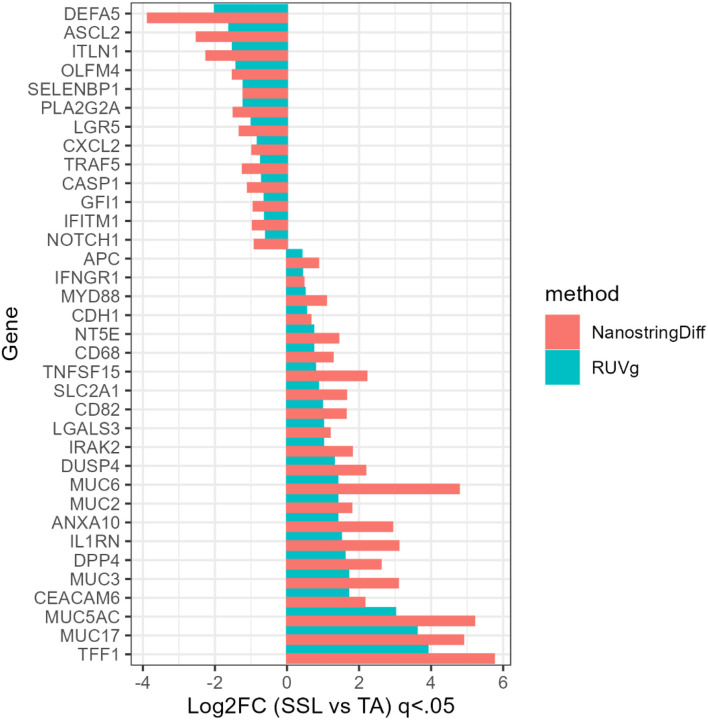
Shared significant (q < 0.05) DEGs for SSLs vs. TAs using the NSDiff and RUVg normalization methods.

**Figure 8:**
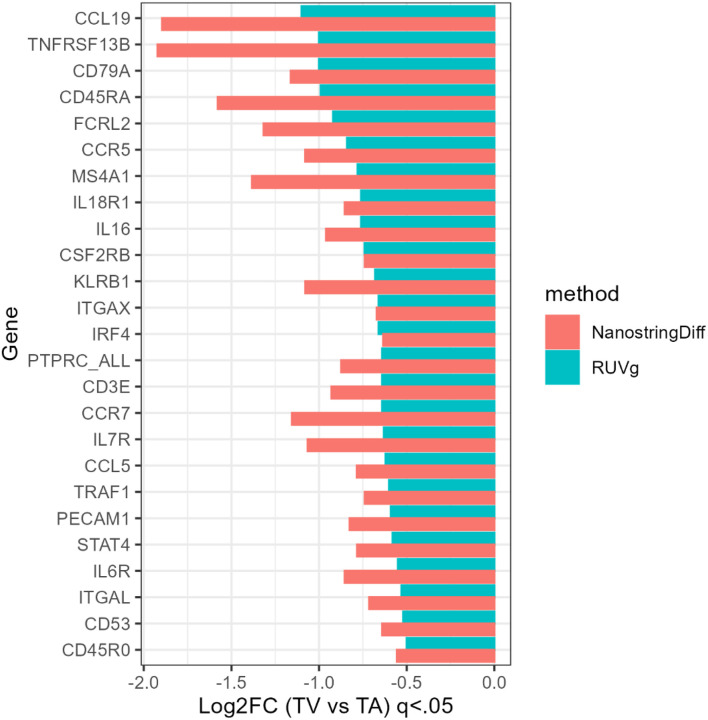
Shared significant (q < 0.05) DEGs for TVs vs. TAs using the NSDiff and RUVg normalization methods.

We identified 35 genes which were labeled as differentially expressed by both NSDiff and RUVg in SSLs vs. TAs (Fig. 7) and 25 shared genes in TVs vs. TAs (Fig. 8). In most cases, the log-fold changes were larger in the NSDiff results than those of RUVg, but always in the same direction. For the SSLs vs. TAs analysis, there were several mucin related genes that were over-expressed in SSLs, such as *TFF1, MUC5AC, MUC17, MUC3, MUC6, ANXA10, CEACAM6, and DUSP4*^23–26^ while genes associated with WNT pathway signaling were more likely under-expressed in the same comparison^23,27–30^ (e.g., *ASCL2, LRG5, OLMF4, ITLN1, PLA2G2A, DEFA5, NOTCH1*). In TVs vs. TAs, all shared genes were under-expressed, depicting a pattern of diminished lymphocytic responses, indicated by lower relative expression of B-cell marker *MS4A1* (*CD20*) and T-cell markers *CD45RA*, *CD3E*, and *CD45RO*. (Fig. 8)

### Type I error

The average empirical Type I error rate (α = 0.05) across the permutation-based simulations were 0.050, 0.114, and 0.053 for RUVg, NSDiff, and nSolver, respectively. This result leads us to conclude that the NSDiff method has a larger potential for making Type I errors over the other two methods.

### Computational complexity

In SSLs vs. TAs (n = 87 samples with 277 genes), RUVg and nSolver were computationally efficient, taking less than 1 second for normalization and DE analysis. In contrast, NSDiff required an average of 96 minutes on a Windows system with 16 GB Ram and an average of 40 minutes on a Windows system with 32 GB of Ram to complete both normalization and DE analysis.

An additional model was adjusted for lesion size, participant age, and sex. The results were not materially different from those of the unadjusted model, so the unadjusted models are presented. We noted that NSDiff required even more computational time with the addition of adjustment variables.

## Discussion

Our quality control (QC) approach, which integrated PCA and a colon-specific positive control gene set, significantly improved sample retention, increasing from 65% in our pilot study using traditional RUV methods to 87% in this study. When comparing three normalization methods and downstream differential expression (DE) analyses, both RUVg and NSDiff labeled significant differentially expressed genes (DEGs) in SSLs and TVs vs. TAs. However, each method had distinct trade-offs. NSDiff, while detecting a broader set of genes, was prone to higher Type I error and was computationally intensive. In contrast, the RUVg approach appropriately controlled Type I error.

Pre-normalization QC is essential for improving the downstream DE analysis by removing outlying samples likely to compromise normalization.^16^ Our pilot study results indicated that the traditional approach – excluding samples with high missingness in endogenous and housekeeping genes – resulted in the exclusion of over one-third of our cases. By incorporating positive control genes recommended by experts into our procedures, we reduced the number of samples excluded by 34%. As others have noted,^17^ positive control genes (e.g. biologic controls) should be endogenous, exhibit a known biologic response, and be differentially expressed under conditions of interest. Our control set meets these criteria: 8 of the 11 control genes were differentially expressed in SSLs vs. TAs, with 4 genes over-expressed (*TFF1, MUC5AC, MUC2, CDH1*) and 4 under-expressed (*ASCL2, ITLN1, OLFM4, SELENBP1*). Moreover, several of the positive control gene set are recognized as potential biomarkers of colon carcinogenesis, supporting the biologic rationale for our 11 gene positive control set.^25–28^

Normalization choice also had a significant impact on DE results. In the comparison between TVs and TAs, RUVg and NSDiff outperformed nSolver, as no significant DEGs were identified by nSolver, suggesting a lack of conservation of biological variability as previously stated by Bhattacharya et al^16^ Given the high likelihood of shared molecular origins between TVs and TAs as part of the MSS pathway^10^ it is not surprising that the gene discrimination between these lesions was more challenging and required more sophisticated normalization approaches. NSDiff is expected to be more powerful than two-stage methods (RUVg), because it directly models count data and jointly normalizes during the DE analysis.^24^ The greater number of DEGs detected with NSDiff suggests it may be more sensitive than RUVg. In the TVs vs. TAs, the shared genes for both RUVg and NSDiff were under-expressed, suggesting a widespread immune suppressive signature in villous containing lesions, with lower expression of T- and B-cell related genes consistent with our earlier work.^8^ At the same time, the discovery potential in the NSDiff approach comes at a cost in terms of computational complexity and occasionally produced inflated log-fold changes, such as −20.81 log2FC for *MARCO* in TVs vs. TAs, or groups of genes with extremely low q-values but tiny log-fold changes (see Fig. 4). This could suggest that the NSDiff method may be less robust, particularly with small sample sizes, as cited in a previous study by Patel et al.^22^

Future research will be needed to validate our 11-gene colon-specific quality control flag and confirm gene expression patterns using a larger sample size. Furthermore, a centralized pathological review will help reduce any misclassification in histologic type, such as identification of mixed histologic types which include both serrated and conventional adenomatous tissue. Our study highlights the value of a tailored gene expression approach for analyzing biologically heterogeneous samples with suboptimal RNA quality. The colon-specific QC flag improved sample retention, while RUVg and NSDiff provided complementary strengths. We recommend leveraging both methods based on study goals: RUVg for reliability and NSDiff for discovery.

## Figures and Tables

**Table 1:** List of colon-specific positive control genes used for the modified QC approach and housekeeping genes used in the standard QC approach.

Codeset	Genes
Colon-specific positive control	*MUC5AC, TFF1, CDH1, CDX2, CTNNB1, ITLN1, B2M, MUC2, OLFM4, PIGR, SELENBP1*
Housekeeping	*ABCF1, ALASI, EEF1G, G6PD, GAPDH, GUSB, HPRT1, OAZ1, POLR1B, POLR2A, PPIA, RPL19, SDHA, TBP, TUBB*

**Table 2:** Distribution of originating institution, age, sex, size, and QC flag status for SSLs, TVs, and TAs.

Variable	Histologic Type			p
	SSL (N = 25)	TV (N = 27)	TA (N = 48)	
**Institution** (%)	10(40)	18(66.7)	34(71)	0.001
Dartmouth	7(28)	1(3.7)	0(0)	
UNC	8(32)	8(29.6)	14(29)	
Vanderbilt				
**Age** (**years**), Mean (SD)	57(8)	58(7)	59(8)	0.62
**Sex** (%)	11(44)	12(44)	18(38)	0.79
Female	14(56)	15(56)	30(62)	
Male				
**Size** (**mm**), Mean (SD)	8.44(4.1)	10.70(6.5)	8.50(3.7)	0.12
**QC Flagged** (%)	4(16)	3(11)	6(12.5)	0.86

## Data Availability

The datasets used and/or analysed during the current study are available from the corresponding author on reasonable request.

## Abbreviations

DE: differential expression
DEG: differential gene expression
MSS: microsatellite stable
NSDiff: NanoStringDiff
PCA: principal components analysis
QC: quality control
RUV: Removal of Unwanted Variation
SSLs: sessile serrated lesions
TAs: tubular adenomas
TVs: tubulovillous and villous adenomas
